# Isolation and identification of flavonoid-producing endophytic fungi from medicinal plant *Conyza blinii* H.Lév that exhibit higher antioxidant and antibacterial activities

**DOI:** 10.7717/peerj.8978

**Published:** 2020-04-15

**Authors:** Zizhong Tang, Yinsheng Wang, Jingyu Yang, Yirong Xiao, Yi Cai, Yujun Wan, Hui Chen, Huipeng Yao, Zhi Shan, Chenglei Li, Gang Wang

**Affiliations:** 1 College of Life Sciences, Sichuan Agricultural University, Ya’an, China; 2 Sichuan Agricultural University Hospital, Ya’an, China; 3 Sichuan Food Fermentation Industry Research and Design Institute, Chengdu, China

**Keywords:** Endophytic fungi, *Conyza blinii* H. Lév, Flavonoids-producing, Antioxidant activity, Antimicrobial activity

## Abstract

**Background:**

*Conyza blinii* H. Lév is a medicinal plant that has a variety of pharmacological activities, but its study is at a standstill due to the shortage of resources.

**Method:**

This study utilized the surface sterilization method to isolate endophytic fungi, and they were preliminarily identified by morphology. Flavonoid-producing strains were screened by NaNO_2_-Al(NO)_3_ colorimetry and further identified by the ITS sequence. Additionally, we used five antioxidant assays (DPPH, Hydroxyl radical, ABTS, FRAP and T-AOC assays) to systematically evaluate the antioxidant capacity of total flavonoids , and we also determined their antibacterial activity.

**Results:**

In this study, 21 endophytic fungi were isolated from wild *Conyza blinii* H. Lév for the first time. There were six flavonoid-producing strains, especially CBL11, whose total flavonoid content reached 50.78 ± 2.4 mg/L. CBL12, CBL12-2 and CBL1-1 all exhibited excellent antioxidant activity. The effect of CBL12 was similar to that of ascorbic acid at low concentrations, and its radical scavenging rates for DPPH and ABTS were 94.56 ± 0.29 % and 99.88 ± 0.27%, respectively, while its IC_50_ values were only 0.11 ± 0.01 mg/mL and 0.2 ± 0.01 mg/mL. Through LC-MS, we found that CBL12 could produce many high-value flavonoids, such as 3-methoxyflavone, nobiletin, formononetin, scopoletin, and daidzein. Additionally, CBL9 had good antibacterial activity against both gram-positive and gram-negative bacteria. Notably, we obtained the high-yield strains CBL12 and CBL9, which not only had high yields (10.64 ± 1.01 mg/L and 10.17 ± 0.11 mg/L, respectively) but also had excellent biological activity. Hence, the results of this study provide new ideas for endophytic fungi that can be exploited as a source of flavonoids and other medicinal components from *Conyza blinii* H. Lév. Moreover, this study can serve as a reference for the development of rare medicinal materials.

## Introduction

Endophytic fungi generally exist in the intercellular space of healthy plant tissues for all or part of their life cycle and have a close symbiotic relationship with the host plant (Arnold, 2007). Moreover, endophytic fungi have significant host specificity and tissue specificity because different habitats will harbor different fungal groups (Unterseher et al., 2013). A wealth of earlier studies suggested that there is an extremely high species diversity among the endophytic fungi, possibly including up to 1 million species (Dreyfuss, 1994), which is a tremendous treasure trove of natural products (Verma, Kharwar & Strobel, 2009). Stierle was the first to isolate an endophytic fungus (*Taxomyces andreanae*) that produced Taxol from *Taxus brevifolia* (Stierle, Strobel & Stierle, 1993). Since then, an increasing number of researchers began to turn their attention to secondary metabolites of endophytic fungi, and more recent studies have certified that most essential secondary metabolites from endophytic fungi that have been reported are important and novel leading compounds, for instance, Taxol, camptothecin (Puri et al., 2005), vinblastine (Kumar et al., 2013), salidroside (Cui et al., 2016), piperine (Chithra et al., 2014), new halogenated dihydroisocoumarins (Zhao et al., 2018), cytotoxic cytochalasins (Kongprapan et al., 2015), podophyllotoxin (Puri et al., 2006) and so on. Recently, endophytic fungi were regarded as a rich source of bioactive metabolites that can be modified to produce desirable novel analogs for medicine, especially as potential anticancer agents (Fathallah et al., 2019). These reports suggested that a growing number of novel natural compounds have been discovered from the endophytic fungi of medicinal plants, which have gradually become a hot spot of research in the fields of agriculture, medicine and industrial research. These results provide a new way to solve the dilemma of resource shortages and low yields of host plants.

*Conyza blinii* H. Lév is a genuine medicinal material in southwestern China, and the whole herb can be used for medicine. It has many secondary metabolites, including flavonoids, terpenoids, saponins, and alkaloids, among which the terpenoids and saponins are the main active components. There are 35 triterpenoid saponins in *Conyza blinii* H. Lév, the majority of which are oleanane-type saponins (Qiao et al., 2010). Moreover, *Conyza blinii* H. Lév has good pharmacological activity with potential anticancer effects (Ma et al., 2017) because its saponin is a dual-targeting autophagy inhibitor for HeLa cells (Liu et al., 2017). It can also protect against acute gastric ulcers because it can efficiently repress inflammatory reactions and protect the gastric mucosa (Ma & Liu, 2014). *Conyza blinii* H. Lév is a biennial plant of the compositae family. However, over-exploitation, a complex growth environment and human activities lead to the destruction of *Conyza blinii* H. Lév as a germplasm resource, and its applications are restricted. Therefore, there have been only a few relevant research studies about *Conyza blinii* H. Lév in recent years, and there are no reports on its endophytic fungi in the literature.

Flavonoids are a class of plant secondary metabolites with a variety of significant pharmacological activities, such as antioxidation, antitumor, anti-inflammatory, antibacterial, and cardiovascular protection (Graf, Milbury & Blumberg, 2005). Because of the molecular structures of polyhydroxyl compounds, flavonoids have good antioxidant biological functions, and for this reason, it has preventive and therapeutic effects against some common diseases (Boudaoud et al., 2009). Therefore, flavonoids have been commercialized as health products with high merit (Xu et al., 2013). There are numerous studies suggesting that endophytic fungi are rich in flavonoids, and some flavonoid-producing endophytic fungi were successfully isolated, such as *Ceriporia lacerata* DMC1106, a new endophytic fungus that can produce the anticancer flavonoid 2′,4′-dihydroxy-6′-methoxy-3′,5′-dimethylchalcone (Wang, Yao & Lu, 2013). The endophytic fungi ST22 and SX10 were obtained from *Ginkgo biloba* L. and can produce flavonoids (Qiu et al., 2010). *Alternaria alternata* SaF-2 and *Fusarium proliferatum* SaR-2 are endophytic fungi from *Salvia miltiorrhiza* that also produce flavonoids, but their yields were only 8.27 and 7.36 µg/mg, respectively (Li et al., 2015). *Epicoccum sorghinum* from *Annona senegalensis* had the highest phenolic content up to 28.85  ± 1.14 mg GAE/g dry weight, and its total antioxidant capacity was 593.46  ± 1.86 µM CRE (Sibanda et al., 2018). Total contents of phenolic compounds and flavonoids in culture filtrate of endophytic *Aspergillus Flavus* L7 were 65.77 mg GAE/mL and 158.33 mg quercetin/mL, respectively (Patil, Patil & Maheshwari, 2015). Pan et al. (2017) found that endophytic fungi from *Fritillaria Unibracteata* produce multifarious natural antioxidant components, including rutin, gallic acid, and phlorizin. The endophytic fungus *Alternaria alternata* AE1 from *Azadirachta indica* A. Juss produces bioactive compounds with strong bactericidal and antioxidant activities, and the GC-MS analysis of the ethyl acetate fraction demonstrated that a number of antioxidant compounds were flavonoids (Chatterjee, Ghosh & Mandal, 2019). With the growth of medicinal value and market perspective, an increasing number of researchers are trying to find a new way to produce more flavonoids. However, as the metabolic production of plants is limited by the environment and spatiotemporal specificity, it is hard to improve the yield of flavonoids from plants, unlike microorganisms (Arisawa & Watanabe, 2017). Therefore, the above results suggested that flavonoid-producing fungi could be regarded as new abundant antioxidant resources with diverse pharmaceutical activities, and we can efficiently produce plentiful flavonoids within a short time as a new method for microbial fermentation to replace plant extraction, which will generate extensive market prospects.

In this study, for the sake of making good use of this rare Traditional Chinese Medicine, we attempted to isolate and identify high-yield flavonoid-producing endophytic fungi from *Conyza blinii* H. Lév and aimed at bridging the research gaps of *Conyza blinii* H. Lév. Furthermore, we used systematic experiments to evaluate the biological activities of their flavonoids and screen active compounds by LC-MS. In this way, we provide a new resource that efficiently produces flavonoids from endophytic fungi that we obtained from *Conyza blinii* H. Lév, instead of directly extracting flavonoids from the rare medicine, as well as effectively facilitating the modernization of *Conyza blinii* H. Lév.

## Materials & Methods

### Experimental material

Healthy samples of *Conyza blinii* H. Lév were collected from Miyi County, Panzhihua City, Sichuan Province, China, in July 2018, were stored in a refrigerator at 4 °C in the laboratory of the Department of Biochemistry and Molecular Biology, Sichuan Agricultural University, and processed as the wild sample within 36 h.

Four bacteria, including *Escherichia coli* (ATCC25922), *Pseudomonas aeruginosa* (ATCC9027), *Bacillus subtilis* (ATCC6633), and *Staphylococcus aureus* (ATCC6538), were used to test the antibacterial activities of the extraction.

### Isolation of endophytic fungi from *Conyza blinii* H. Lév

The leaves and stems were washed with running water and immersed in distilled water until the samples were saturated and restored. First, the tissue masses were immersed in 75% ethanol solution for 1 min, followed by 4% sodium hypochlorite solution for 3 min. Then, they were rinsed with sterile distilled water 3–4 times. Next, the surface-sterilized parts were cut into 1 cm × 1 cm tissue masses with a sterile scalpel and inoculated onto potato dextrose agar (PDA) medium with 50 mg/L ampicillin (AMP). Finally, these plates were incubated in a 28 °C constant temperature incubator for 7–9 days. Meanwhile, to ensure the fungi isolated from this experiment were the endophytic fungi of *Conyza blinii* H. Lév, two control groups were set up. In one group, the disinfected tissue was directly inoculated on the medium without cutting, and in another group, the last rinse solution was spread on the medium. If both control groups grew without any microorganisms, we could be sure that the fungi we obtained were the endophytic fungi of *Conyza blinii* H. Lév.

When hyphae grew out at the edge of the tissue wound, different hyphae were inoculated separately into fresh PDA culture medium (AMP) and placed in a 28 °C constant temperature incubator for 5–7 days until the hyphae were separated and stored at 4 °C.

### Preliminary morphological identification of endophytic fungi from *Conyza blinii* H. Lév

We observed and recorded the growth rate of hyphae, the morphology of colonies and pigment production. Microscopic identification was carried out by the cover slip culture method, which could determine the characteristics of the different hyphae. A sterilized cover slip was obliquely inserted into the growth edge of the colony. When the hyphae were attached to the cover slip, the cover slip was removed from the medium. Then, conidial structure and a hyphal septum were observed under a 10 × 40 field of vision (microscope model: OLYMPUS CX23) that was recorded by a camera. Finally, these fungi were classified through a fungal identification manual.

### Detection of total flavonoids in the fermentation broth of endophytic fungi

Twenty-one endophytic fungi were inoculated into 100 mL of fresh PDA liquid medium (200 g/L leachate of fresh potato, 20 g/L glucose, pH 7.0) and then cultured on a shaker for 7 days (160 rpm/min and 28 °C).

After fermentation, the liquid was filtered out and extracted twice with 25 mL of ethyl acetate. After that, the extracted liquid was collected and concentrated by vacuum rotary evaporation until the product became dry. Then, methanol was added up to 5 mL, and the solution was filtered with a 0.22 µm syringe filter.

The total flavonoid content of ethyl acetate was evaluated by NaNO_2_-Al(NO)_3_ colorimetry, which was used for generating a rutin standard curve. First, a 20 mg rutin standard was dissolved with 100 mL of 60% ethanol to obtain the mother liquor, and 2.5 mL of the solution was pipetted into 2.5 mL of 60% ethanol. Eventually, the standard solution concentration was 0.1 mg/mL and diluted to a gradient of 6 concentrations. In each test tube, 60 µL of one of six different concentrations of the standard solution was added, followed by 20 µL of 5% NaNO_2_, standing for 6 min, the addition of 20 µL of 10% Al(NO_3_)_3_, standing for 6 min, and the addition of 1 mol/L NaOH. Finally, a solution of 60% ethanol was utilized to reach a volume of 200 µL, followed by standing for 15 min, and the absorbance of the mixture was measured at 510 nm (UV spectrophotometer model: UNICO, UV-3802).

The fermentation broth concentration was determined according to the standard system. All the steps were the same as those above, and each group had 3 parallel controls. The flavonoid content of each sample was calculated with reference to the rutin standard curve.

Total flavonoid content = (mass concentration × dilution ratio × methanol solution volume)/total volume of fungal fermentation broth × 100%.

### Molecular biological identification of flavonoid-producing endophytic fungi

The fungal genome DNA was extracted by the SDS method, and the universal primers ITS1 and ITS4 were used to amplify the internal transcribed spacer (ITS) region (5.8S rRNA gene) of these fungi by PCR. The reaction product was detected by agarose gel electrophoresis, and when the fragment sizes were all between 500 bp and 750 bp (DNA Marker: Solarbio D2000), the results showed that ITS sequence amplification was successful. Then, the PCR products were recovered, and the purified DNA samples were sequenced by Invitrogen/Thermo Fisher Scientific (Shanghai).

After successful sequencing, the DNA sequences were compared by BLAST, similar sequences were downloaded, and a phylogenetic tree alignment was performed with MEGA 7.0 software by the neighbor-joining method.

### Antioxidant activity of total flavonoids from endophytic fungal crude extraction

PDA liquid medium was used for fermenting the 6 flavonoid-producing endophytic fungi for 7 days under the same conditions (160 rpm/min, 28 °C), and the fermentation was extracted twice with ethyl acetate according to the method in 2.4. The extraction was performed through vacuum rotary evaporation until the product became dry; 5 mL of absolute ethanol was added to dissolve the sample and then filtered with a 0.22 µm syringe filter.

The prepared solution was freeze-dried and adjusted to 1 mg/mL. The abovementioned 6 kinds of fermentation broth and the positive control (ascorbic acid) were adjusted to five concentrations (0.0 mg/mL, 0.2 mg/mL, 0.4 mg/mL, 0.6 mg/mL, 0.8 mg/mL, 1.0 mg/mL) for five antioxidant activity assays (1,1-diphenyl-2-picrylhydrazyl (DPPH) free radical scavenging, 2,2′-azino-bis(3-ethylbenzothiazoline-6-sulfonic acid) (ABTS) radical scavenging, hydroxyl radical scavenging, Fe^3+^ reducing power, and total antioxidant capacity assays) and stored in a freezer at −20 °C. SPSS 23.0 was used for statistical analyses.

#### Detection of DPPH radical scavenging activity

The DPPH radical has a single unpaired electron that results in strong absorption at 517 nm, while its alcohol solution is purple. When a free radical scavenger is present, its absorption gradually disappears due to the pairing of its single electron, which could be widely used in the analysis of antioxidant capacity (Mensor et al., 2001).

DPPH powder (25 mg) was accurately weighed and dissolved in 50 mL of anhydrous ethanol. Then, it was transferred to a 100 mL volumetric flask and diluted to a constant volume. Finally, a 0.25 mg/mL DPPH-ethanol solution was obtained and stored in a 4 °C refrigerator.

Then, 100 µL of DPPH solution and 100 µL of gradient sample solution were added to an enzyme label plate and mixed on the basis of the concentration gradient, and the sample solution was incubated in the dark for 30 min at room temperature (24 °C). The OD value was measured at 517 nm (Enzyme-labeled instrument model: Thermo Scientific, Multiskan Sky) with anhydrous ethanol as the blank control, and each experimental group had 3 parallel controls.

(1) \begin{eqnarray*}Y(\text{%})= \left[ 1- \frac{{A}_{1}-{A}_{2}}{{A}_{0}} \right] \times 100\text{%}\end{eqnarray*}

*Y* is the DPPH clearance rate, A_0_ is the absorbance of 100 µL of DPPH solution plus 100 µL of anhydrous ethanol, A_1_ is the absorbance of 100 µL of DPPH solution mixed with 100 µL of gradient sample solution, and A_2_ is the absorbance of 100 µL of gradient sample solution plus 100 µL of anhydrous ethanol.

#### Detection of ABTS radical scavenging activity

ABTS is oxidized to green ABTS ⋅ + with appropriate oxidants, and the production of ABTS ⋅ + is inhibited in the presence of antioxidants (Re et al., 1999).

ABTS powder (78 mg) was accurately weighed and added to 13.2 mg of potassium persulfate into 20 mL of ultrapure water at 4 °C, which was stored in the dark for 16 h. After stabilization, the solution was stored as an ABTS stock solution. Before the ABTS radical scavenging experiment, the ABTS stock solution was diluted with anhydrous ethanol to an absorbance of 0.7  ± 0.02. Then, 2 mL of ABTS solution was mixed with 100 µL of gradient sample solution and incubated for 6 min. Finally, 200 µL of supernatant was aspirated and added to an ELISA plate. The absorbance was measured at 734 nm using anhydrous ethanol as a blank control, and each experimental group had 3 parallel controls.

(2) \begin{eqnarray*}Y(\text{%})= \left[ 1- \frac{{A}_{1}-{A}_{2}}{{A}_{0}} \right] \times 100\text{%}\end{eqnarray*}

*Y* is the clearance rate of ABTS, A_0_ is the absorbance of ABTS solution mixed with anhydrous ethanol, A_1_ is the absorbance of ABTS solution mixed with the sample, and A_2_ is the absorbance of the sample mixed with anhydrous ethanol.

#### Detection of hydroxyl radical scavenging ability

Hydroxyl radicals have a strong ability to acquire electrons, and hydroxyl radical scavenging activity is a vital indicator of antioxidant activity. We used the Fenton reaction to assay this activity (Chimi et al., 1991).

First, 1,10-phenanthroline monohydrate (5 mmol/L), PBS (phosphate-buffered saline) buffer (pH = 7.4, 0.2 mol/L), ferrous sulfate (7.5 mmol/L), and H_2_O_2_ (0.1%) solutions and a sample concentration gradient of 0∼1.0 mg/mL were prepared for this experiment. The reaction system was as follows: 75 µL of 1,10-phenanthroline monohydrate solution, 200 µL of PBS buffer solution, and 500 µL of each gradient sample in sequence. The sample was mixed, and then 50 µL of ferrous sulfate solution and 100 µL of H_2_O_2_ solution were added and mixed; the sample was incubated in a 37 °C water bath for 30 min. The absorbance was measured at 510 nm with 200 µL samples added to the ELISA plate, and each experimental group had 3 parallel controls.

(3) \begin{eqnarray*}Y= \frac{{A}_{1}-{A}_{0}}{{A}_{2}-{A}_{0}} \end{eqnarray*}

*Y* is the clearance rate of the hydroxyl group, A_1_ is the absorbance of a reaction solution containing the sample solution and H_2_O_2_, A_0_ is the absorbance of a blank solution with the sample solution replaced with anhydrous ethanol, and A_2_ is the absorbance of a solution with distilled water instead of H_2_O_2_.

#### Detection of Fe^3+^-reducing power

Fe^3+^-reducing antioxidant power (FRAP) assays measure the antioxidant capacity of samples using ferrous ions and TPTZ (tripyridyltriazine) to form a blue-violet complex at low pH. They are widely used in the analysis of antioxidant capacity (Benzie & Strain, 1996).

First, 250 µL of extract solution, 250 µL of PBS (pH = 6) and 250 µL of 1% potassium ferricyanide solution were added to an Eppendorf tube, vortexed, and then placed in a 50 °C water bath for 20 min. At this time, the solution was removed and cooled to room temperature (24 °C), and 250 µL of 10% trichloroacetic acid (TCA) solution was added; the mixture should not be exposed to light during this step. Thereafter, the sample was centrifuged at 3,000 rpm for 10 min, and then 300 µL of supernatant was aspirated and added to a solution containing 300 µL of distilled water and 60 µL of 0.1% FeCl_3_. The solution was then allowed to sit for 10 min. The sample was added to an ELISA plate, and its absorbance was measured at 700 nm. Each experimental group had 3 parallel controls.

(4) \begin{eqnarray*}N={A}_{1}-{A}_{2}\end{eqnarray*}

The absorbance indicates the reducing power, N is the reducing power of Fe, A_1_ is the absorbance of the supernatant mixed with distilled water and FeCl_3_ solution, and A_2_ is the absorbance of supernatant mixed with distilled water.

#### Detection of total oxidation resistance of T-AOC

The following reaction system (Table 1) was constructed in an Eppendorf tube according to a kit for determining total antioxidant capacity (T-AOC) produced by Nanjing Jiancheng Company, China.

**Table 1:** Reaction system 1 of T-AOC.

**Solution name**	**Tube**	**A test tube used as a control**
Reagent 1 application solution (mL)	1.0	1.0
Test sample (mL)	0.1	0
Reagent 2 application solution (mL)	2.0	2
Reagent 3 application solution (mL)	0.5	0.5

Samples were fully mixed by a vortex generator, placed into a 37 °C water bath for 30 min, and then removed and cooled with cold water immediately. Then, another system (Table 2) was added and was mixed completely. The sample was allowed to sit for 10 min, and ddH_2_O was set as the blank control. The absorbance of each tube was measured at 520 nm with a light beam diameter of 1 cm. A total antioxidant capacity unit was defined as a milliliter of sample increasing the absorbance (OD) value by 0.01 per minute at 37 °C. Each experimental group had 3 parallel controls.

(5) \begin{eqnarray*}Y= \frac{{A}_{1}-{A}_{0}}{0.01} + \frac{30\times 3.2}{0.1} \end{eqnarray*}

*Y* represents the antioxidant capacity of total flavonoids, A_0_ is the absorbance of the test tube used as a control, and A_1_ isthe absorbance of the test tube.

**Table 2:** Reaction system 2 of T-AOC.

**Solution name**	**Tube**	**A test tube used as a control**
Reagent 3 application solution (mL)	0.1	0.1
Test sample (mL)	0	0.1

### Antibacterial activity of total flavonoids from endophytic fungal crude extraction

Six flavonoid endophytic fungi were inoculated in 100 mL of fresh PDA liquid medium and cultured at 28 °C and 160 rpm for 7 days. The fermentation broth was then removed, and the filtrate was extracted with 25 mL of ethyl acetate. Ethyl acetate was evaporated to dryness with vacuum rotary evaporation, and the sample was dissolved by adding 5 mL of methanol. Then, the solution was stored in a freezer at −20 °C.

Four bacteria (*Escherichia coli*, *Pseudomonas aeruginosa*, *Bacillus subtilis*, and *Staphylococcus aureus*) were activated by 10 mL of LB medium and cultured at 37 °C, then shaken at 180 rpm for 12 h. Next, the sterilized LB liquid was used to dilute the bacterial solution 1,000 fold.

The antibacterial activity was detected by the cylinder-plate method. A bacterial solution was applied to LB solid medium, and three Oxford cups were placed as experimental groups. Then, 200 µL of 10 mg/mL ampicillin was added as a positive control, 200 µL of methanol was added as a negative control, and 200 µL of the sample was added. Three parallel groups were set in each group. After incubation in a 37 °C constant temperature incubator for 12 h, the corresponding inhibition zone sizes were observed and recorded.

### Preliminary identification of the flavonoids from CBL12 by LC-MS

A test sample of the flavonoids from CBL12 was prepared according to the method described above, which obtained the ethyl acetate part via freeze-drying as the test sample. The sample was sent to the Novogene Company (Beijing, China) for the non-targeting LC-MS metabolomic test.

## Results

### Isolation and identification of endophytic fungi from *Conyza blinii* H. Lév

In our study, 21 different endophytic fungi were isolated from the leaves and stems of *Conyza blinii* H. Lév, comprising 12 strains from the leaves and 9 strains from the stems, and the corresponding endophytic fungi were numbered in order. The endophytic fungi isolated from *Conyza blinii* H. Lév are shown in Table 3.

**Table 3:** The separation result of endophytic fungi from *Conyza blinii* H. Lév.

**Plant issue**	**Strain number**	**Total**
Leaves	CBL1 CBL1-1 CBL2 CBL4 CBL4-1 CBL5-2 CBL6 CBL7 CBL9 CBL11 CBL12 CBL12-2	12
Stems	CBS1 CBS1-1 CBS2 CBS4 CBS6 CBS8 CBS9 CBS10 CBS12	9

### Preliminary morphological identification of endophytic fungi from *Conyza blinii* H. Lév

**Figure 1:**
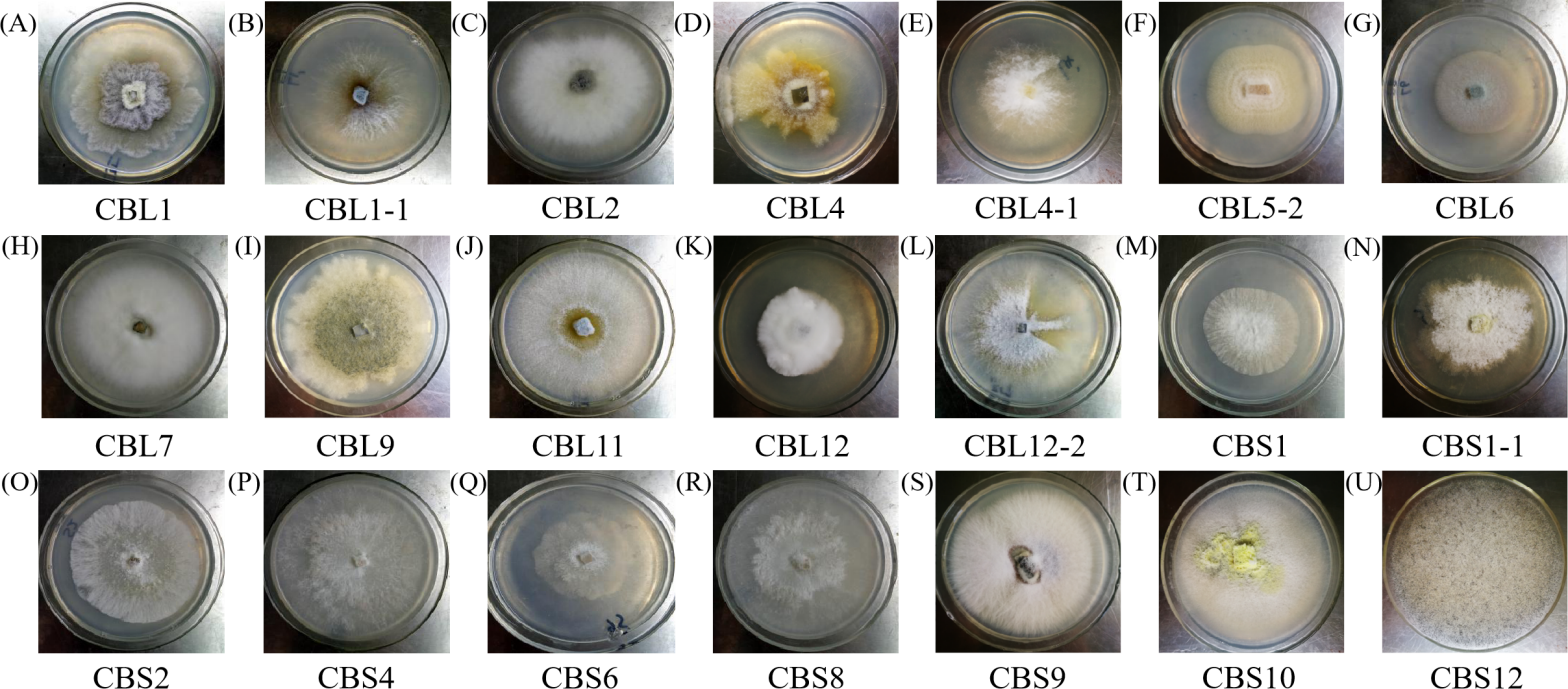
The colony morphology of endophytic fungi *Conyza blinii* H. Lév. A-U are the 21 different endophytic fungi, respectively.

Different endophytic fungi were cultured in fresh PDA medium, and then the morphological characteristics of each colony were recorded and observed to determine whether any pigment was produced. Pictures of each fungus colony are shown in Fig. 1.

**Figure 2:**
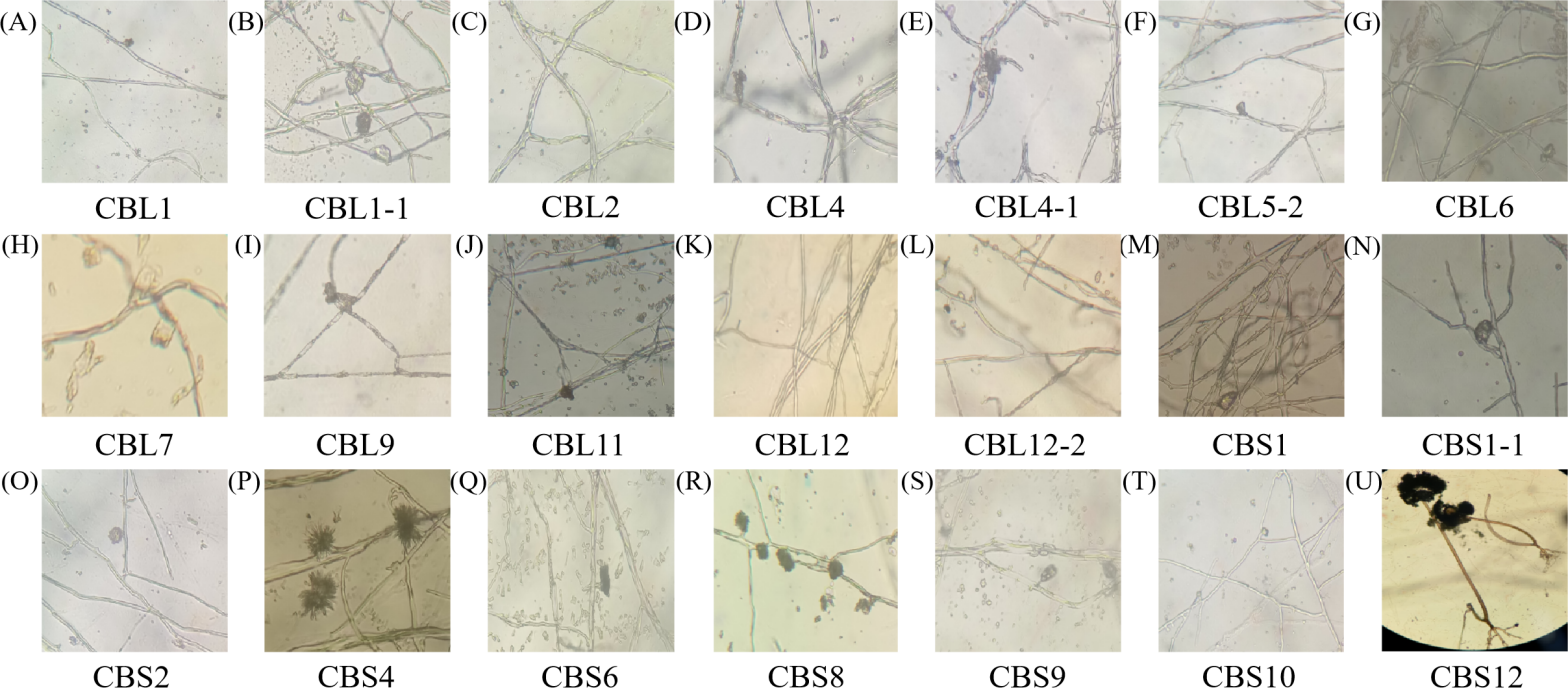
The shape characteristics of hypha. A–U are the characteristics of hypha with 21 parts, respectively.

The morphology of hyphae and spore structures was observed with a microscope under a 10 × 40 field of vision. Combining the data with the previously determined morphological characteristics of the colonies, it can be concluded that endophytic fungi of *Conyza blinii* H. Lév mainly belong to *basidiomycetes*, *deuteromycota*, *ascomycota* and *rhizopus*, which indicated that there were diverse endophytic fungi isolated from *Conyza blinii* H. Lév (Fig. 2).

### Screening the flavonoid-producing endophytic fungi

**Figure 3:**
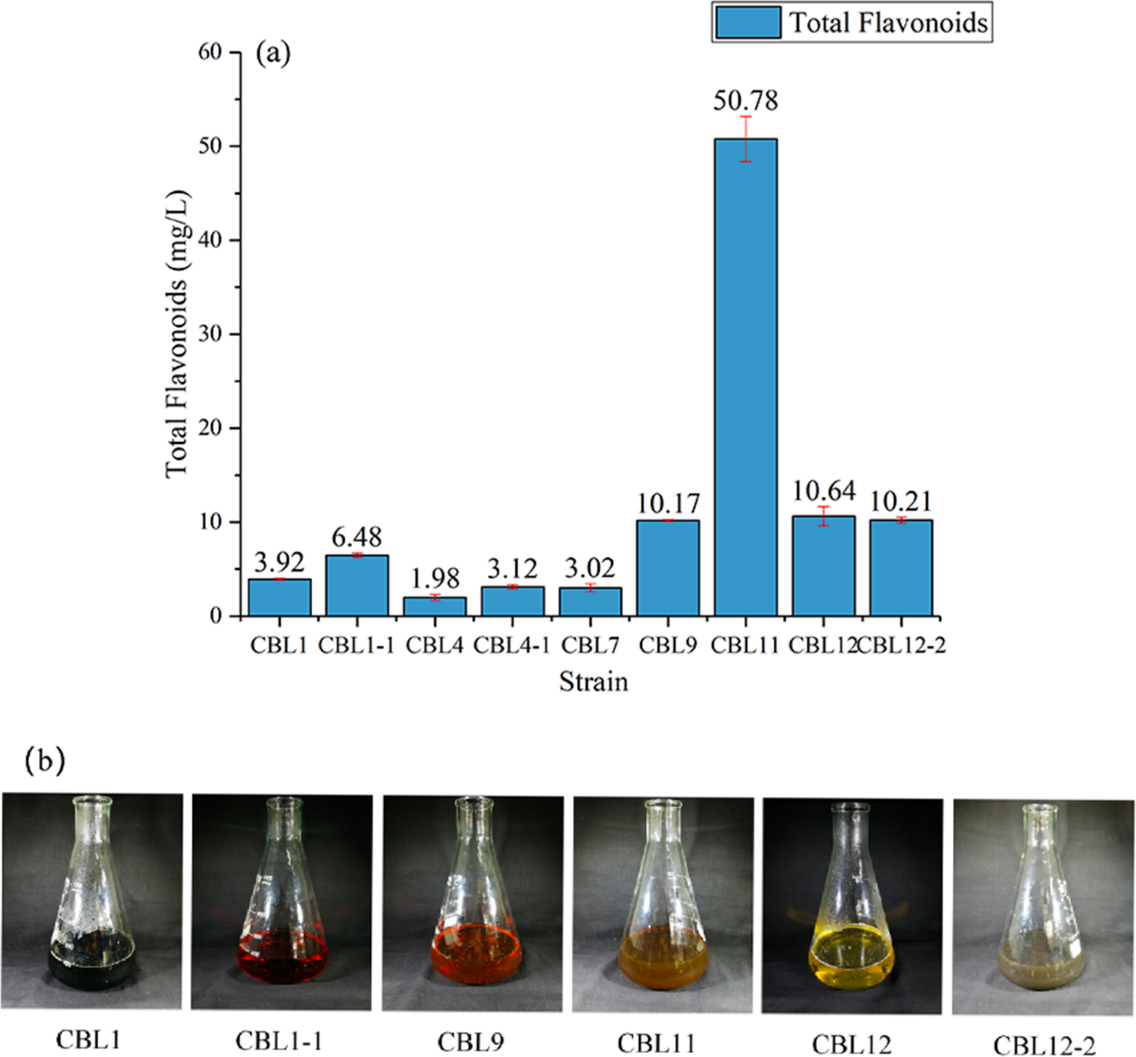
Screening the flavonoids-producing endophytic fungi. (A) Determination of total flavonoids in the fermentation broth of endophytic fungi. (B) The fermentation broth of flavonoids-producing endophytic fungi.

The quantitative analysis of flavonoids in fermentation broth was established by a rutin standard curve (*R*^2^ = 0.998). Then, on this basis, the total flavonoid contents from 21 different fermentation broths were determined by NaNO_2_-Al(NO_3_)_3_ colorimetry, and the results suggested that six strains had the potential to produce flavonoids (Fig. 3A). Among them, the strain CBL11 produced a high yield of 50.78 ± 2.4 mg/L, and another three strains (CBL9, CBL12, CBL12-2) reached approximately 10 mg/L, which means they reached a high yield level. Figure 3B shows the characteristics of flavonoid-producing endophytic fungal fermentation broth after filtration.

### Molecular identification of the flavonoid-producing endophytic fungi

**Figure 4:**
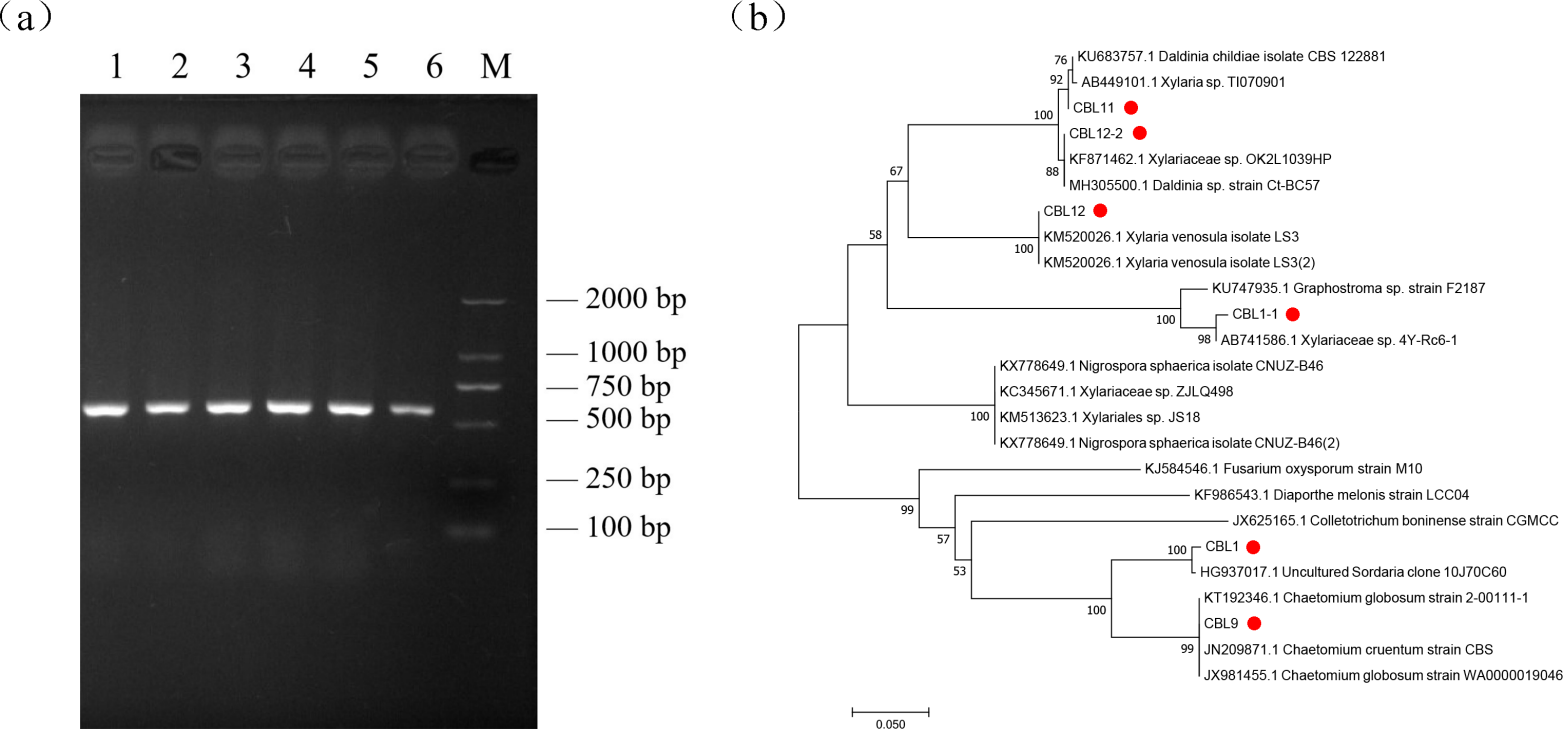
The results of molecular biological identification. (A) Amplification of ITS sequences, M is Marker: D2000. 1–6 are CBL1 to CB L12-2 in order, (B) Phylogenetic tree analysis of flavonoids-producing endophytic fungi from *Conyza blinii* H. Lév.

Genomic DNA was extracted by the SDS method and used for PCR, and the ITS sequences of endophytic fungi were verified by agarose gel electrophoresis (AGE). The results showed that all fragments were between 500 bp and 750 bp in length, which proved that ITS sequence amplification was successful, and the DNA marker was D2000 (Fig. 4A).

Sequencing the obtained ITS sequence was performed by Invitrogen (Shanghai, China); subsequently, a homologous sequence alignment of ITS sequences was uploaded to NCBI BLAST for comparison to obtain relevant homologous strain and evolution information. The results are shown in Table 4. The results showed that the major flavonoid-producing endophytic fungi belong to *Chaetomium globosum* and a species of *Xylariaceae.* After that, the sequence information of 10 strains with the best aligned sequences was downloaded, and MEGA7.0 software was used for phylogenetic tree analysis to further analyze the relationships of these fungi. Combining phylogenetic tree and previous morphological identification, we can further determine the species of the flavonoid-producing endophytic fungi from *Conyza blinii* H. Lév (Fig. 4B).

**Table 4:** The homologous strain information of flavonoids-producing endophytic fungi.

**Strain number**	**Highly homologous strain**	**Sequence coverage**	**Consistency**	**E-value**	**Accession number**
CBL1	*Uncultured sordaria*	100%	98%	0.0	HG937017.1
	*Uncultured cercophora*	100%	98%	0.0	GU055523.1
CBL1-1	*Xylariaceae sp.*	99%	99%	0.0	AB741586.1
CBL9	*Chaetomium cruentum*	99%	99%	0.0	JN209871.1
	*Chaetomium globosum*	99%	99%	0.0	HQ914911.1
CBL11	*Daldinia childiae*	98%	99%	0.0	KU683757.1
	*Daldinia pyrenaica*	98%	99%	0.0	KY610413.1
CBL12	*Xylaria venosula*	99%	100%	0.0	KM520026.1
	*Fungal endophyte isolate*	99%	100%	0.0	KY038605.1
CBL12-2	*Xylariaceae sp.*	99%	99%	0.0	KF871462.1
	*Hansfordia sp.*	97%	99%	0.0	HG971771.1

### Antioxidant activity of total flavonoids from endophytic fungal crude extraction

**Figure 5:**
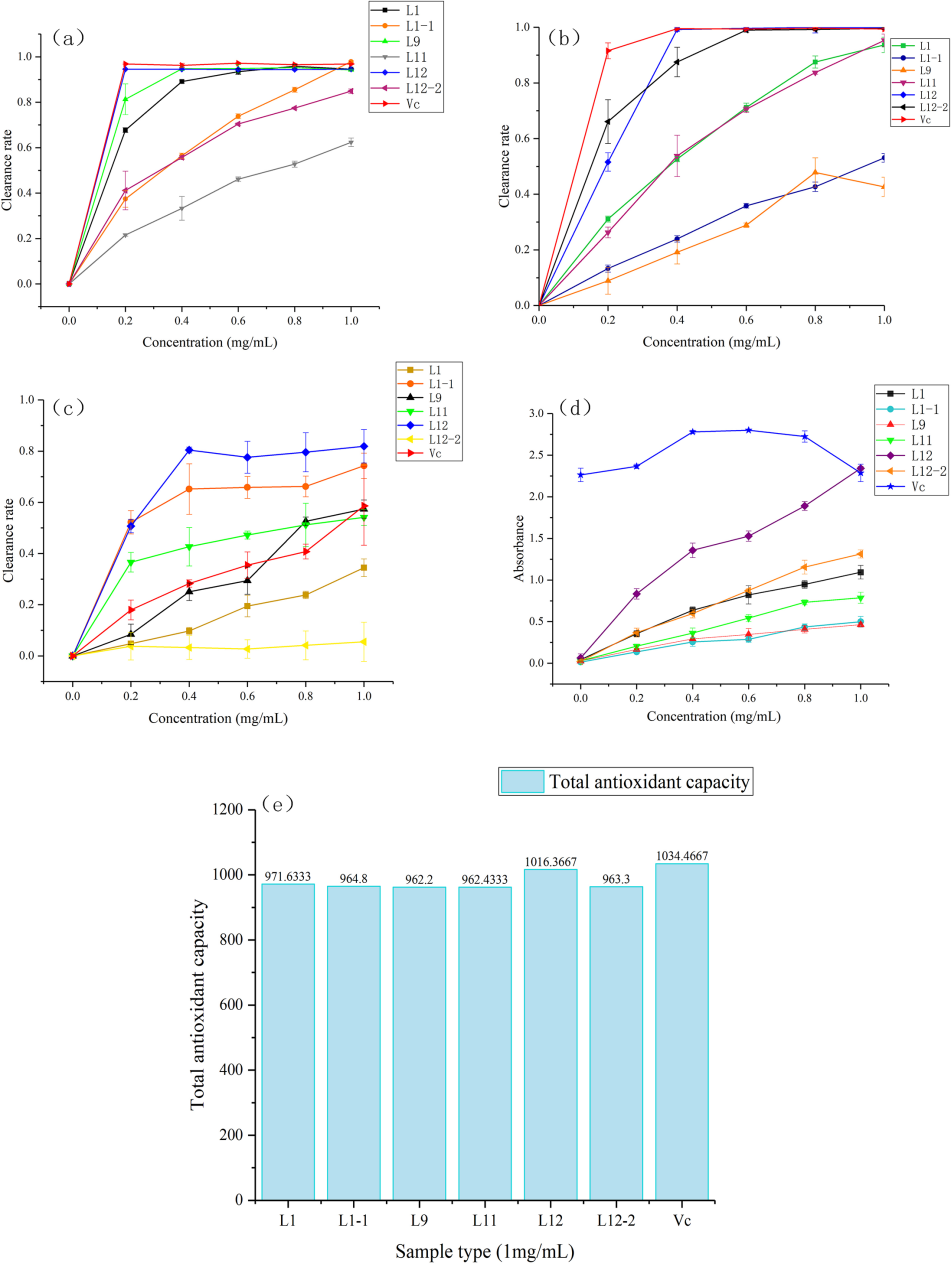
Six samples through freeze drying to compound with five gradient concentrations (0.0 mg/mL, 0.2 mg/mL, 0.4 mg/mL, 0.6 mg/mL, 0.8 mg/mL, 1.0 mg/mL) , use the ascorbic acid (Vc) as the control. (A) Activity of DPPH radical scavenging. (B) Activity of ABTS radical scavenging. (C) Activity of OH ⋅ radical scavenging. (D) Reducing power of Fe^3+^. (E) Total antioxidant capacity of T-AOC.

All samples within the concentration range of 0∼1.0 mg/mL scavenged DPPH free radicals, and this activity was positively correlated with the sample concentration. The DPPH radical scavenging rates of each sample at the highest concentration (1.0 mg/mL) followed the descending order of CBL1-1 (97.89 ± 0.38%), ascorbic acid (96.86 ± 0.20%), CBL12 (94.56 ± 0.29%), CBL1 (94.55 ± 0.47%), CBL9 (94.24 ± 0.30%), CBL12-2 (84.86 ± 1.01%) and CBL11 (62.36 ± 1.88%). Moreover, at the lowest concentration (0.2 mg/mL), scavenging abilities of CBL12, CBL1, and CBL9 for DPPH free radicals were almost the same as that of the positive control (ascorbic acid) (Fig. 5A). Their IC_50_ values were 0.11 ± 0.01 mg/mL (CBL12), 0.14 ± 0.01 mg/mL (CBL1), 0.15 ± 0.03 mg/mL (CBL9), and 0.10 mg/mL ascorbic acid (*P* < 0.05, *n* = 3).

The results also showed that the ABTS radical scavenging rates of each sample at the highest concentration (1.0 mg/mL) followed the descending order of CBL12 (99.88 ± 0.27%), CBL12-2 (99.63 ± 0.34%), ascorbic acid (99.40 ± 0.29%), CBL11 (95.30 ± 2.27%), CBL1 (93.69 ± 2.79%), CBL1-1 (53.08 ± 1.60%), and CBL9 (42.63 ± 3.48%). At 0.4 mg/mL, the ABTS radical scavenging rate of CBL12 was basically equal to that of ascorbic acid (Fig. 5B). The IC_50_values of these samples were 0.2 ± 0.01 mg/mL (CBL12), 0.13 ± 0.06 mg/mL (CBL12-2), and 0.11 mg/mL (ascorbic acid) (*P* < 0.05, *n* = 3).

Hydroxyl free radical scavenging activity was observed in all samples in the concentration range of 0∼1.0 mg/mL, and the hydroxyl radical scavenging rates of each sample at the highest concentration (1.0 mg/mL) followed the descending order of CBL12 (81.89 ± 6.53%), CBL1-1 (74.28 ± 4.95%), ascorbic acid (58.60 ± 1.53%), CBL9 (57.36 ± 3.56%), and CBL11 (54.13%). The effects of CBL12 and CBL1-1 were obviously better than that of ascorbic acid (Fig. 5C). Additionally, the IC_50_values of the active samples were 0.19 ± 0.02 mg/mL (CBL12), 0.19 ± 0.03 mg/mL (CBL1-1), and 0.96 ± 0.06 mg/mL (ascorbic acid) (*P* < 0.05, *n* = 3). These results fully demonstrate that the flavonoids from CBL12 and CBL1-1 exhibit excellent antioxidant activity.

The statistical analysis of each IC50 value is shown in Table S1. These results suggested that there was a significant difference between the experimental group and ascorbic acid.

In the FRAP experiment, when the ascorbic acid concentration was 0.6 mg/mL, the reducing power was the highest, and the sample’s absorbance was 2.800. The Fe^3+^-reducing power of other samples increased with increasing sample concentration. The absorbance values of each sample at the highest concentration (1.0 mg/mL) followed the descending order of CBL12 (2.346 ± 0.023), ascorbic acid (2.288 ± 0.010), CBL12-2 (1.313 ± 0.049), and CBL1 (1.093 ± 0.081) (*P* < 0.05, *n* = 3) (Fig. 5D).

The T-AOC of each sample followed the descending order of ascorbic acid, CBL12, CBL1, CBL1-1, CBL12-2, CBL11, and CBL9, while the T-AOC of CBL12 was almost as same as that of ascorbic acid (Fig. 5E).

Through these five antioxidant experiments, we can easily summarize that the flavonoid-producing endophytic fungi have extremely high antioxidant activity associated with their flavonoids and have excellent prospects. The flavonoids from strain CBL12, whose activity was similar to that of ascorbic acid, have good medical implications and deserve further research.

### Antibacterial activity of total flavonoids from endophytic fungal crude extraction

**Figure 6:**
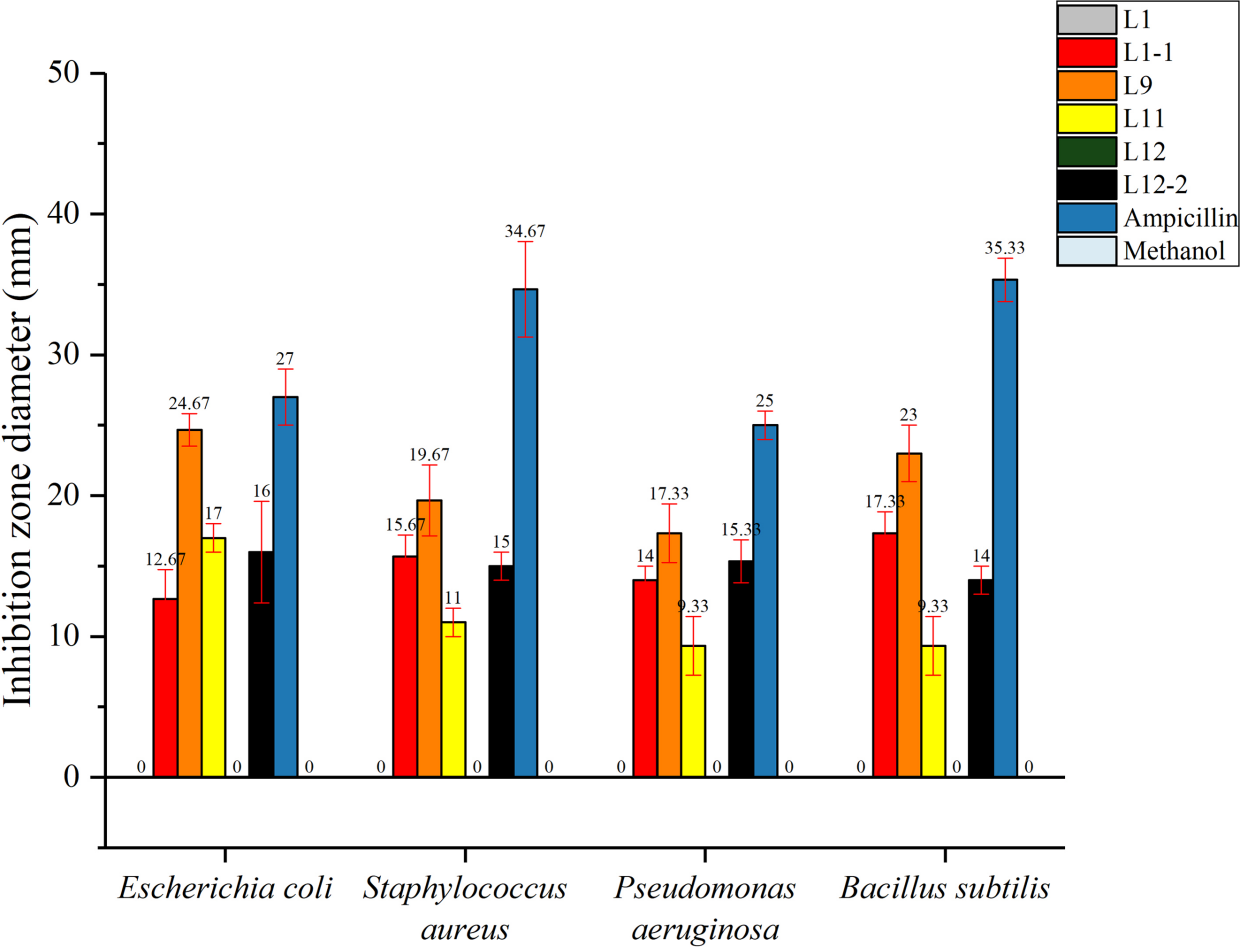
The antibacterial activity of six flavonoid samples. The Ampicillin, methyl alcohol were be as the control. The assay bacterium are two Gram-positive bacteria: *Staphylococcus aureus, Bacillus subtilis*, and two Gram-negative bacteria: *Escherichia coli, Pseudomonas aeruginosa*.

In this study, the total flavonoids from CBL9 had efficient antibacterial activity that inhibited both gram-positive and gram-negative bacteria. Among them, CBL9 had the best effect on *Escherichia coli*, with an inhibition zone diameter of up to 24.67 ± 1.15 mm. Other inhibition zone diameters of CBL9 were as follows: *S. aureus* (19.67 ± 2.52 mm), *Bacillus subtilis* (23 ± 2 mm), and *Pseudomonas aeruginosa* (17.33 ± 2.08 mm) (*P* < 0.05). (Fig. 6). In addition, the total flavonoid fermentation broth from CBL12-2, CBL11, and CBL1-1 exhibited antibacterial activity, and CBL9 has the potential for development into an antibacterial treatment.

Ampicillin and methanol were set as positive and negative controls, respectively, and each experimental group had 3 parallel controls.

### Preliminary identification of the flavonoids from CBL12 by LC-MS

Because of the excellent antioxidant activity of CBL12, we used LC-MS to preliminarily identify the flavonoids from CBL12. The results showed that there were various flavonoids produced by the endophytic fungus CBL12, including multiple high-value flavonoids, such as 3-methoxyflavone, nobiletin, formononetin, scopoletin, daidzein, etc. The representative compounds and specific information are shown in Table 5.

**Table 5:** Preliminary identification of the flavonoids from CBL12 by LC-MS.

Name	Formula	Molecular Weight	RT [min]	Annotation Source: ChemSpider Search	Annotation Source: mz Cloud Search	Annotation Source: mz Vault Search	ChemSpider Results	Mz Cloud Best Match	Mz Vault Best Match	0007318-1A
3-Methoxyflavone	C_16_H_12_O_3_	252.07813	9.936	No results	Full match	Full match	0	75.8	58.4	5205055.918
Nobiletin	C_21_H_22_O_8_	402.12999	11.989	No results	Full match	Full match	0	79.4	70.3	19949007.14
Formononetin	C_16_H_12_O_4_	268.07298	9.848	No results	Full match	Full match	0	77.3	68.2	13380009.6
Scopoletin	C_10_H_8_O_4_	192.04218	8.52	No results	Full match	Full match	0	75.9	62.9	86537928.17
Daidzein	C_15_H_10_O_4_	254.05741	9.833	No match	Full match	Full match	1	81.7	79.4	7156010.818
Tangeritin	C_20_H_20_O_7_	372.1198	12.565	No results	Full match	Full match	0	86.9	64.7	32307468.43
Fisetin	C_15_H_10_O_6_	304.05722	7.462	No results	Invalid mass	Invalid mass	0	83.2	66.3	10902954.69
Trioxsalen	C_14_H_12_O_3_	228.07815	9.954	No results	Full match	Full match	0	70.8	61.8	6138572.408
D-(+)-Maltose	C_12_H_22_O_11_	364.09688	1.323	No results	Invalid mass	Invalid mass	0	91.4	78.7	28267582.08
Hesperetin	C_16_H_14_O_6_	302.07808	8.536	No results	Full match	No results	0	68.9		16274872.97
Sakuranetin	C_16_H_14_O_5_	286.08339	9.918	No results	Full match	No results	0	50.8		57180036.68
Catechin	C_15_H_14_O_6_	290.07842	9.843	No results	Full match	No results	0	69.7		12395753.47
3-Hydroxyflavone	C_15_H_10_O_3_	238.06228	9.942	No results	No results	Full match	0		73.2	10118970.97
Isoliquiritigenin	C_15_H_12_O_4_	256.07317	8.336	No results	Full match	No results	0	63.2		11845660.09
(-)-Epigallocatechin	C_15_H_14_O_7_	306.07292	5.964	No results	Full match	No results	0	84.2		1862682.909
3-Methoxycinnamic acid	C_10_H_10_O_3_	160.05228	10.315	No results	Invalid mass	No results	0	84.3		147475434.9

## Discussion

In this study, a total of 21 endophytic fungi were isolated from *Conyza blinii* H. Lév for the first time. Endophytic fungi from medicinal plants produce a large number of biologically active secondary metabolites due to the interactions between endophytic fungi and the host plant, so we isolated endophytic fungi with rich secondary metabolites that have excellent biological activity from *Conyza blinii* H. Lév. Because endophytic fungi have host specificity and tissue specificity and are relevant to the plant environment (AbreuTarazi et al., 2010), we isolated wild endophytic fungi samples from an optimal growth environment (Miyi County, Sichuan, China).

We screened 6 strains of flavonoid-producing endophytic fungi that belong to *Xylariales*, *Chaetomium globosum*, and *Daldinia childiae*. In particular, *Xylariales* is one of the most significant fungal sources of secondary metabolites and produces lead pharmaceutical compounds (Helaly, Thongbai & Stadler, 2018). *Chaetomium globosum* and *Daldinia childiae* are also important sources of natural products (Qin et al., 2010; Zhao et al., 2017), so the endophytic fungi we isolated had good value for research and application. The strain CBL11 had the highest yield with a total flavonoid content up to 50.78 ± 2.4 mg/L. In previous literature, the total flavonoid content has generally not been high. Qiu et al. (2010) isolated two flavonoid-producing endophytic fungi and their flavonoid yield was only 11.43 mg/L and 12.56 mg/L. According to the results of the current study, the yield of flavonoid-producing endophytic fungi was relatively low and cannot meet market needs. However, as the medicinal value of flavonoids is increasing, we need to search for endophytic fungi with higher flavonoid yields and better antioxidant activity.

Previous studies have shown that free radicals are harmful to human health and are always mediators of disease and tissue injury (Kehrer & Klotz, 2015), and flavonoids possess good antioxidant capacity that can scavenge free radicals. Thaipong et al. conducted four experiments (hydroxyl radical scavenging, ABTS scavenging, Fe^3+^ reducing power and T-AOC assays) to set a good standard for evaluating the antioxidant capacity of samples (Thaipong et al., 2012). In this study, we combined 5 different *in vitro* antioxidant experiments to indicate that the flavonoid from CBL12 had excellent antioxidant activity; its effects were much better than those in previous studies, and it showed great potential for exploration. Uma Anitha & Mythili (2017) analyzed the antioxidant potential of the endophytic fungus *Achaetomium* sp. From *Euphorbia hirta*, and the DPPH clearance rate of total flavonoids ranged from 66.890 ± 1.385% to 87.340 ± 0.289%. In this study, not only did CBL12 have a flavonoid yield of up to 10.64 ± 1.01 mg/L but also its DPPH radical scavenging assay rate was up to 94.55 ± 0.12%, (0.2 mg/mL) with an IC_50_ of only 0.11 ± 0.01 mg/mL, which was similar to ascorbic acid at an equivalent concentration. Furthermore, according to the LC-MS results, CBL12 had the potential to produce abundant high-value flavonoids, so there was no doubt that it will have broad prospects as a resource for natural antioxidant agents and promote more medicinal research of *Xylariales*. For instance, based on our study, these flavonoids can be produced by microbial fermentation, allowing economical production of these compounds on an industrial scale (Wu et al., 2014), which can avoid cumbersome extraction from plants.

Meanwhile, the results of a recent study suggested that flavonoids also have significant antibacterial activity due to their function of inhibiting several bacterial virulence factors (Alurappa et al., 2014). In our study, the CBL9 sample had inhibitory effects on both gram-positive bacteria and gram-negative bacteria, with inhibition zone diameters reaching up to 24.67 ± 1.15 mm. It can be seen that the flavonoid-producing endophytic fungi from *Conyza blinii* H. Lév exert high yield and good biological activity, providing a basis for the further study of endophytic fungal metabolites of *Conyza blinii* H. Lév. Interestingly, compared with previous research, CBL12 and CBL9 have not only high total flavonoid contents (10.64 ± 1.01 mg/L, 10.17 ± 0.11 mg/L) but also excellent biological activity. This meant that the two flavonoid-producing endophytic fungi have better application prospects for medicine and will promote research on endothytic fungi from rare medicinal plants.

Plant accumulation of secondary metabolites has spatiotemporal specificity (Kong et al., 2017), and the expression of synthetic genes is strictly regulated by the growth environment (Lepelley et al., 2007), so it is difficult for us to directly extract the target secondary metabolites from plants. Relative to plants, microorganisms have a simpler metabolic network and more easily process genetic modification (Levskaya et al., 2005), leading us to use microorganisms to produce natural products efficiently. All in all, we can use endophytic fungi to produce a high amount of valuable flavonoids and provide a new way for extracting these compounds, not only from plants, which will promote the exploitation and application of flavonoids.

In addition, as endophytic fungi have a close relationship with their host plant, some endophytes can produce the same compound as the host plant (Chen et al., 2010), impact plant fitness and defense response (Yan et al., 2019), promote plant growth (Wang et al., 2005) and affect the accumulation of secondary metabolites in the host. Zhai et al. (2017) found that a *Salvia miltiorrhiza* endophytic fungus (*Chaetomium globosum* D38) significantly enhances the accumulation of tanshinone and salvianolic acid by the host. Moreover, this study combined rare medicinal materials with endophytic fungi to give excellent guidance for the modernization of Traditional Chinese Medicine, providing a new way to explore the high-value compounds from these insufficient sources and promote related research. This is the first study on the endophytic fungi of *Conyza blinii* H. Lév, which increases the knowledge in this field, and we find that these endophytic fungi can produce high-value flavonoids. We also hope that we can find additional novel compounds from these sources in the future and will continue to explore whether the fungi can accelerate *Conyza blinii* H. Lév growth or expedite the accumulation of secondary metabolites.

## Conclusions

In this study, 21 strains of endophytic fungi were isolated from the rare medicinal plant *Conyza blinii* H. Lév for the first time, and via morphology identification, the species were preliminarily determined. We obtained six flavonoid-producing strains, and among them, strain CBL11 had the highest yield, reaching up to 50.78 ± 2.4 mg/L, which was significantly higher than other strains previously reported. ITS sequence analysis indicated that the six strains mainly belong to *Chaetomium globosum* and *Xylariaceae*. Then, we used five experiments (DPPH, hydroxyl radical, ABTS, FRAP and T-AOC assays) to systematically evaluate the antioxidant capacity of total flavonoids in fermentation broth. The results showed that CBL12, CBL12-2 and CBL1-1 all had excellent antioxidant activity; remarkably, the effect of CBL12 was similar to that of ascorbic acid at low concentrations (0.2 mg/mL), and its scavenging rates of DPPH, ABTS, and hydroxyl radicals were 94.56 ± 0.29%, 99.88 ± 0.27%, and 81.89 ± 6.53%, respectively. The IC_50_ values were only 0.11 ± 0.01 mg/mL, 0.2 ± 0.01 mg/mL, and 0.13 ± 0.06 mg/mL, respectively. Notably, LC-MS results suggested that there were various flavonoids produced by the endophytic fungus CBL12, including multiple high-value flavonoids, such as 3-methoxyflavone, nobiletin, formononetin, scopoletin, and daidzein. In addition, CBL9 showed good antimicrobial activity; specifically, it was found to inhibit both gram-positive and gram-negative bacterial growth, with inhibition zone diameters reaching 24.67 ± 1.15 mm for *Escherichia coli* and 23 ± 2 mm for *Bacillus subtilis*.

This study was the first to isolate endophytic fungi from wild *Conyza blinii* H. Lév and screen the high-flavonoid strain CBL11; the high-total flavonoid strain CBL12, with excellent antioxidant activity; and the strain CBL9, with antibacterial activity. Furthermore, this work compensates for the absence of research on *Conyza blinii* H. Lév, provides a new idea for the development of rare medicinal materials, and lays the foundation for the drug exploitation of *Conyza blinii* H. Lév.

##  Supplemental Information

10.7717/peerj.8978/supp-1 Table S1 The statistical analysis of IC_50_ value

10.7717/peerj.8978/supp-2 Data S1 Original LC-MS data of CBL12

10.7717/peerj.8978/supp-3 Data S2 Raw data

## Additional Information and Declarations

### Data Availability

The following information was supplied regarding data availability: The raw measurements are available in the Supplemental Files.
