# Supplementary material for: Isolation and identification of flavonoid-producing endophytic fungi from medicinal plant Conyza blinii H.Lév that exhibit higher antioxidant and antibacterial activities
Source: PeerJ. 2020 Apr 15;8:e8978. doi: 10.7717/peerj.8978 (PMC7166047; doi:10.7717/peerj.8978)
Supplement: Table S1 [file peerj-08-8978-s001.docx]

**Table.S1** The statistical analysis of IC50 value

|  | DPPH | Hydroxyl radical | ABTS |
| --- | --- | --- | --- |
| CBL1 | 0.1447±0.0057 a | **1.3467±0.0950 c | **0.3613±0.0110 b |
| CBL1-1 | **0.3203±0.0216 b | **0.1857±0.0264 a | **0.9413±0.0211 c |
| CBL9 | *0.1506±0.0315 a | 0.8313±0.0222 b | **1.1433±0.0945 d |
| CBL11 | **0.7013±0.0342 c | 0.7920±0.3124 b | **0.3780±0.0338 b |
| CBL12 | 0.1057±0.0001 a | **0.1913±0.0184 a | 0.1297±0.0632 a |
| CBL12-2 | **0.2937±0.0414 b | 1.0457±0.0774 b | 0.1186±0.0762 a |
| Ascorbic acid | 0.1032±0.0003 a | 0.9446±0.0554 b | 0.1093±0.0034 a |

Note: ’*’ represent P<0.05, have significant difference; ’**’ represent P<0.01, have most significant difference; with no ’*’ represent have no significant difference (compare with ascorbic acid)。Different letters represent a similar subset at 0.05 significance level ( P<0.05, n=3).
